# Tuning the Mechanical and Thermal Properties of Hydroxypropyl Methylcellulose Cryogels with the Aid of Surfactants

**DOI:** 10.3390/gels7030118

**Published:** 2021-08-11

**Authors:** Rafael S. Dezotti, Laíse M. Furtado, Márcio Yee, Ticiane S. Valera, Krishnasamy Balaji, Rômulo A. Ando, Denise F. S. Petri

**Affiliations:** 1 Fundamental Chemistry Department, Institute of Chemistry, University of São Paulo, Lineu Prestes 748, São Paulo 05508-000, SP, Brazil; rafael.dezotti@alumni.usp.br (R.S.D.); laise_furtado@usp.br (L.M.F.); raando@iq.usp.br (R.A.A.); 2 Marine Science Department, Federal University of São Paulo, Carvalho de Mendonça 144, Santos 11070-100, SP, Brazil; marcio.yee@unifesp.br; 3 Metallurgical and Materials Engineering Department, Polytechnic School, University of São Paulo, Mello Moraes 2463, São Paulo 05508-030, SP, Brazil; tsvalera@usp.br; 4 Polymer Engineering Laboratory, PSG Institute of Technology and Applied Research, Neelambur, Coimbatore 641062, India; balaji.psgtech@yahoo.co.in

**Keywords:** cryogels, hydroxypropyl methylcellulose, AOT, kolliphor, compressive modulus, thermal conductivity, microstructure

## Abstract

The mechanical and thermal properties of cryogels depend on their microstructure. In this study, the microstructure of hydroxypropyl methylcellulose (HPMC) cryogels was modified by the addition of ionic (bis (2-ethylhexyl) sodium sulfosuccinate, AOT) and non-ionic (Kolliphor^®^ EL) surfactants to the precursor hydrogels (30 g/L). The surfactant concentrations varied from 0.2 mmol/L to 3.0 mmol/L. All of the hydrogels presented viscous behavior (G″ > G′). Hydrogels containing AOT (c > 2.0 mmol/L) led to cryogels with the lowest compressive modulus (13 ± 1 kPa), the highest specific surface area (2.31 m^2^/g), the lowest thermal conductivity (0.030 W/(m·°C)), and less hygroscopic walls. The addition of Kolliphor^®^ EL to the hydrogels yielded the stiffest cryogels (320 ± 32 kPa) with the lowest specific surface area (1.11 m^2^/g) and the highest thermal conductivity (0.055 W/(m·°C)). Density functional theory (DFT) calculations indicated an interaction energy of −31.8 kcal/mol due to the interaction between the AOT sulfonate group and the HPMC hydroxyl group and the hydrogen bond between the AOT carbonyl group and the HPMC hydroxyl group. The interaction energy between the HPMC hydroxyl group and the Kolliphor^®^ EL hydroxyl group was calculated as −7.91 kcal/mol. A model was proposed to describe the effects of AOT or Kolliphor^®^ EL on the microstructures and the mechanical/thermal properties of HPMC cryogels.

## 1. Introduction

Porous polymeric matrices are important for developing new biological devices (drug delivery, scaffolds for cells) and thermal and sound insulating materials. There are different methods for preparing 3D porous polymeric structures. One of them is the addition of porogenic agents to the polymerization process or to the polymer system. The choice of porogens requires a careful analysis on their chemical structure, physical state, solubility, and miscibility with other chemical constituents (monomer, initiator, solvent) [1]. Other strategies are based on solvent removal from the polymer gel precursors. For instance, aerogels are gels in which the pores are filled with air. The most traditional method to obtain them is by exchanging the gel solvent with supercritical CO_2_; after decreasing the pressure, CO_2_ molecules change from their liquid state to their gas state, creating pores in the polymeric structures [2,3,4]. Cryogels are formed after defrosting the frozen precursor gel. In the case of hydrogels, upon freezing, the ice crystals expand and compress the polymer chains. The removal of ice crystals through freeze-drying often yields macropores (pore size above 50 nm) in the spongy polymeric structure [2,3,4]. On the other hand, exchanging water with *tert*-butanol in the precursor gels might generate mesopores (pore size between 2 nm and 50 nm) in the structure because the crystallization of *tert*-butanol does not involve expansion [5].

Polysaccharide-based porous structures are sustainable materials because they stem from renewable sources, they are biodegradable and nontoxic, and their preparation does not generate hazardous waste. Aerogels and cryogels have interconnected micro- and macropores and low density and low thermal conductivity, respectively. Recently, Zou and Budtova presented an overview on the correlation between the thermal conductivity, density, and surface area of polysaccharide-based aerogels and cryogels reported in the literature [6]. They concluded that low thermal conductivity values require porous materials with a density of ~0.1 g cm^−3^ and surface area larger than several hundreds of m^2^ g^−1^. For instance, chitosan aerogels presented a thermal conductivity of 0.022 W/(m K) and a specific surface area of 545 of m^2^ g^−1^ [7]. Pectin-TiO_2_ based aerogels presented a thermal conductivity of (0.022–0.025 W m^−1^ K^−1^) [8], whereas aeropectin presented a thermal superinsulating property (0.016–0.025 W m^−1^ K^−1^) and density values below 0.15 g cm^−3^ [9]. However, for cellulose-based cryogels and aerogels, the correlation between low thermal conductivity and high density or high surface area was not constant [6]; the discrepancies might be due to the cellulose source and/or to the method of preparation [10].

One strategy to control the microstructure of cryogels and therefore their properties is the addition of surfactants to the hydrogel precursor. Ni et al. prepared Konjac glucomannan-based cryogels from hydrogel precursors containing different glyceryl monostearate contents [11]. A high content of glyceryl monostearate led to a more uniform ice crystals and therefore to a more homogeneous pore size as well. The concentration of polysiloxane-based surfactants also altered the structure of polyurethane foams due to liquid–gas interfacial effects during cell growth [12]. Poly (alkoxy) triblock surfactants combined with ethyl ether were used as porogens for the preparation of the porous structure of poly (ethylene glycol methyl ether acrylate-*co*-polyethylene glycol diacrylate) [13]. However, systematic studies on the effect of different types and concentrations of surfactants on the cryogel microstructure (average pore size, surface area) and consequently on the thermal conductivity and mechanical properties are sparse. 

Hydroxypropyl methylcellulose (HPMC) is a cellulose ether with amphiphilic character controlled by the degree of substitution (DS) of the methyl groups and the molar substitution (MS) of the hydroxypropyl groups in the repeated unit [14]. Due its versatility, HPMC plays an important role as a thickener, emulsifier, binder, and film former in the formulations of food, drug, and construction products [15]. HPMC cryogels are prepared by irradiating frozen hydrogels containing photoinitiators with UV light [16] and by esterification with citric acid [17]. In this study, we hypothesized that the addition of Kolliphor^®^ EL (Polyoxyl-35 castor oil), a non-ionic surfactant with long alkyl chains that is frequently used as emulsifier in cosmetic and drug formulations [18], or dioctyl sulfosuccinate salt sodium (AOT), a double-chained anionic surfactant with short alkyl chains that is generally used in reverse micelle systems [19], to HPMC hydrogel precursors would impact the microstructure of the resulting cryogels. Different contents of each type of surfactant led to cryogels, which were analyzed using scanning electron microscopy (SEM), X-ray microtomography (micro CT), compression tests, and thermal conductivity and thermogravimetic analyses. The interactions between Kolliphor or AOT and HPMC were evaluated by density functional theory (DFT) calculations, which supported the experimental observations. This study is relevant because it demonstrates an easy strategy to tune the microstructure and consequently the properties of cryogels.

## 2. Results and Discussion

The sample codes and their compositions are listed in Table 1. Table 2 shows the physical properties determined for the cryogel samples after rinsing to remove unreacted molecules and freeze-drying. The mean apparent density ranged from 31 ± 4 kg/m^3^ (AOT5) to 39 ± 5 kg/m^3^ (K7.25). The gel content, which indicates the efficiency of the esterification reaction between citric acid carboxyl groups and HPMC hydroxyl groups, decreased from 86 ± 1% (HPMC~K0.5) to 61 ± 1% (K7.25) as the Kolliphor^®^ EL concentration in the precursor hydrogels increased. This finding indicates that the Kolliphor^®^ EL hydroxyl groups might compete with the HPMC hydroxyl groups for esterification with the citric acid carboxylic acid groups [20]. In the case of samples containing AOT, no significant change in the gel content values was observed. Infrared vibrational spectra (Figure S1) did not show evidence of the presence of surfactants in the cryogel structures; the low surfactant content and the band overlap did not allow for any specific identification. However, the presence of the band at 1729 cm^−1^ assigned as the ester -C=O stretching vibrations in all of the cryogels evidenced crosslinking between HPMC and citric acid [21,22]. Noteworthy is that the spectrum of pure HPMC presented the typical polysaccharide bands [21,22], but no band at ~1729 cm^−1^.

**Figure 1:**
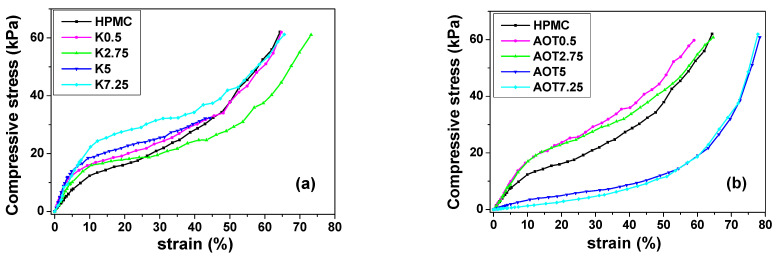
Compressive tests of cryogels containing (**a**) Kolliphor^®^ EL and (**b**) AOT under the strain rate of 0.010 s^−1^ at 23.7 ± 0.7 °C and a relative air humidity of 61 ± 4%, *n* = 10.

Figure 1 shows typical compression stress–strain curves determined for HPMC (control), K0.5, AOT0.5, K2.75, AOT2.75, K5, AOT5, K7.25, and AOT7.25 cryogels. Table 2 shows the values of the Young’s modulus (E) determined in the linear elastic region (from 0 to 5% strain), where Hook’s laws is valid [23], and the yield stress (σ_Y_) at which the material starts to plastically deform for all of the cryogels. All of the cryogels prepared with Kolliphor^®^ EL were considerably stiffer than pure HPMC cryogels. On the other hand, the addition of a low content (less than 1.0 mM) of AOT in the hydrogel precursors resulted in the cryogels being slightly stiffer than HPMC cryogels, whereas the addition of a higher content (more than 2.0 mM) of AOT yielded very soft cryogels. AOT7.25 presented a mean E value one order of magnitude smaller than that of the HPMC cryogel. A plausible explanation for this effect is that the addition of AOT yielded cryogels with thinner cell walls than those of pure HPMC cryogels.

**Figure 2:**
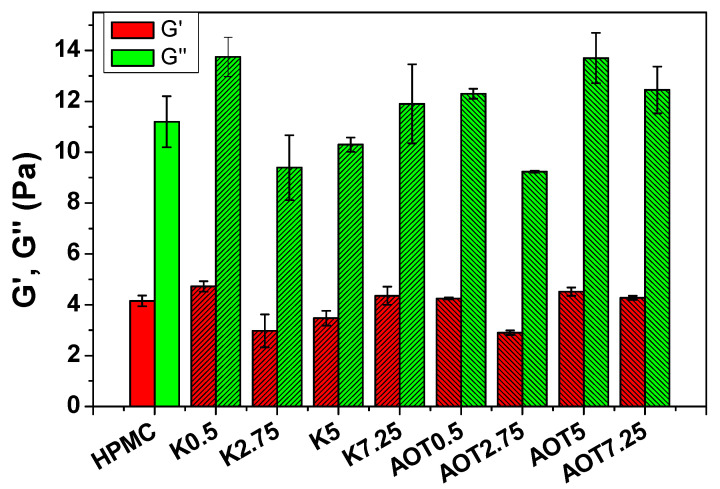
G′ and G″ obtained for precursor hydrogels by SAOS tests at a frequency of 0.1 Hz and a strain amplitude of 0.5%.

The type or the amount of surfactant had no substantial effect on the rheological behavior of the precursor hydrogel at 25.0 ± 0.5 °C. All of the hydrogels showed viscous behavior (G″ > G′) (Figure 2), in agreement with reported studies for HPMC hydrogels at the similar concentration and temperature [24]. Table 2 shows the damping factor (tan δ) = (G″/G′) of the precursor hydrogels obtained by SAOS at 0.1 Hz; all values were similar, regardless of the composition. Therefore, one can conclude that there is no correlation between the rheological behavior of the precursor hydrogels and the mechanical properties of the resulting cryogels. Furthermore, the surfactants did not significantly affect the flow behavior of the HPMC chains in the hydrogels. However, upon freezing and/or freeze-drying the hydrogels, the presence of Kolliphor^®^ EL stiffened the HPMC cell walls, whereas the presence of AOT (at concentration higher than the cmc) yielded softer cryogels.

**Figure 3:**
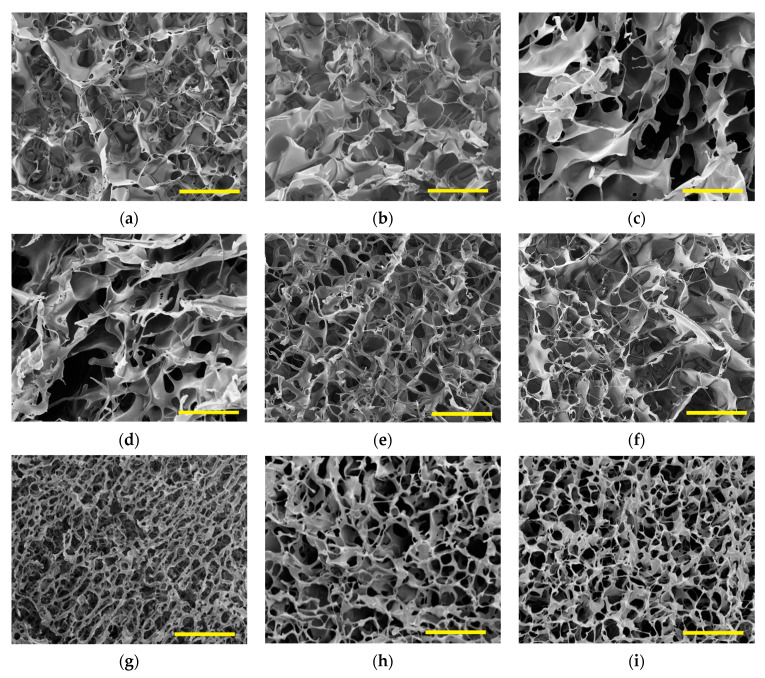
SEM images of (**a**) HPMC, (**b**) K0.5, (**c**) K2.75, (**d**) K5, (**e**) K7.25, (**f**) AOT0.5, (**g**) AOT2.75, (**h**) AOT5, and (**i**) AOT7.25. The scale bars correspond to 200 µm.

**Figure 4:**
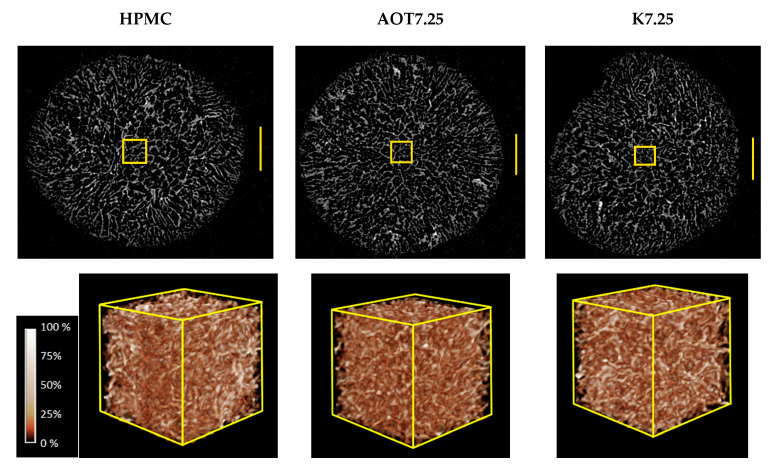
Reconstructed 3D models from microtomography data of HPMC, AOT7.25, and K7.25 cryogel samples. The top images correspond to transversal slices of the total diameter (ø = 5 mm), and the scale bale corresponds to 1 mm. The cubes (500 µm × 500 µm × 500 µm) correspond to the central region of the samples; the attenuation scale was obtained using the transfer function in the software CTVox^®^. Black and white correspond to the minimal and maximal X-ray attenuation, respectively.

Figure 3 shows the scanning electron microscopy (SEM) images obtained for the cryogels. All of the cryogels presented open cell structures. The irregular shape of the pores hindered a reliable quantitative analysis of the pore size distribution. Qualitatively, the cryogels prepared in the presence of Kolliphor^®^ EL presented large pores in a lesser amount, implying cell wall thickening. On the other hand, the cryogels prepared with AOT presented small pores in larger quantities, implying cell wall thinning. These trends explain the high values of Young’s moduli determined for cryogels prepared with Kolliphor and the low values of Young’s moduli determined for cryogels prepared with AOT (Table 2). Figure 4 shows typical reconstructed 3D models from microtomography data of HPMC, AOT7.25, and K7.25 cryogel samples. The analyses of the volumes of interest (VOIs) yielded the specific area $(A_{sp}$), connectivity density (the average number of cell wall connections in 1 mm^3^ calculated by “Conneulor” algorithm [25]), and average pore size presented in Table 3. The pore size distributions in the HPMC, K7.25, and AOT7.25 samples calculated with CTan^®^ software are provided in Figure S2.

Table 3 shows that the AOT7.25 cryogels presented the highest values of connectivity density and surface area and the smallest average pore size corroborating the trends observed in the SEM images (Figure 3). The surface areas of ~2.3 m^2^ g^−1^ and ~1.8 m^2^ g^−1^ determined for AOT and HPMC are of the same order of magnitude of those determined for cellulose nanofibrils/nonionic polyoxamer-based foams [26] and nanofibrillated cellulose composite cryogels [27,28]. Furthermore, AOT7.25 and HPMC cryogels presented similar connectivity density values, consistent with the similar gel content values presented in Table 2. The K7.25 cryogels presented the lowest connectivity density and surface area values and the largest average pore size, in agreement with the lowest gel content (Table 2) and the SEM images, respectively. The low connectivity density might be explained by the fact that the Kolliphor^®^ EL hydroxyl groups compete with the HPMC hydroxyl groups for esterification with citric acid. One should notice that the connectivity density correlates to the amount of the connected polymeric material to form the cell walls [29]. Cryogels tend to present low surface area because the polymer chains are compressed by ice crystal growth, which increases the pore size. Thus, the presence of Kolliphor aggregates with a typical size of 12–15 nm [30] might increase the size of the ice crystals, increasing the mean pore size in the resulting cryogel. Furthermore, during the freezing process, growing crystals will expel Kolliphor molecules, so the surfactant and HPMC will concentrate in the non-frozen liquid zone, which is where phase separation can take place.

**Figure 5:**
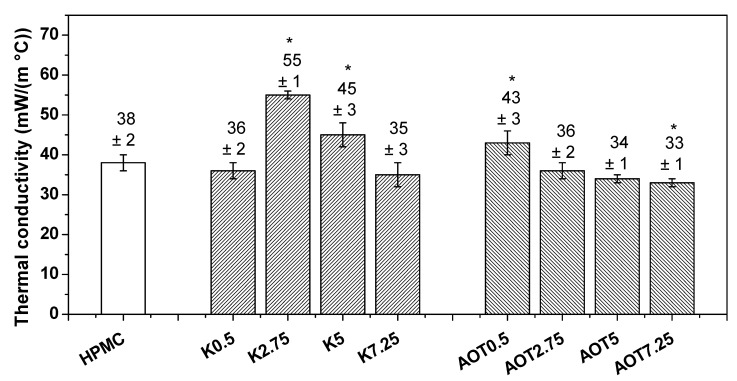
Thermal conductivities determined for all cryogels at 23.7 ± 0.7 ° C and 60% relative humidity. The thermal conductivity of dry air was 25 mW m^−1^ °C^−1^. “*” stands for data statistically different from HPMC with *p* < 0.05.

Figure 5 shows the thermal conductivity (*k*) values obtained using the THS method at 23.7 ± 0.7 °C and 60% relative humidity. The linear region between 46 s and 164 s of each curve was fitted to linear regression (Figure S3); the lowest R^2^ value obtained was 0.98520. AOT7.25 presented the lowest *k* value (0.033 W m^−1^ °C^−1^) although there was no statistically significant difference (*p* ≤ 0.05, *n* = 3) among this *k* value and those determined for the AOT2.75, AOT5, K0.5, and K7.25 cryogels; it was statically different from the HPMC (0.038 W m^−1^ °C^−1^) cryogel. Cellulose nanofibrils crosslinked with 1,2,3,4-butanetetracarboxylic acid and *N*-methylol dimethylphosphonopropionamide led to sponges with a *k* value (0.0326 W m^−1^ °C^−1^) and an E value of ~5 kPa [28]; these properties are similar to those determined for the AOT7.25 *k* value (0.033± 0.001 W m^−1^ °C^−1^) and E value of ~13 ± 1 kPa. The highest *k* values were observed for K2.75 (0.055 W m^−1^ °C^−1^), K5 (0.045 W m^−1^ °C^−1^), and AOT0.5 (0.043 W m^−1^ °C^−1^); similar values were reported for nanofibrillated cellulose sponges [26,28]. Materials with a large number of small pores tend to present lower thermal conductivity than those with large pores. The addition of surfactants above their critical micelle concentration might favor the formation of small pores, as shown by Mondal and Khakhar [31], because the possible interfacial tensions in the system are reduced. 

The *k* values obtained are consistent with the range observed by Jimenez-Saelices et al. [32]. The heat transport in foams can be described by the contribution of four factors, as shown in Equation (1):

(1) $$k_{total}=k_{sol}+k_{gas}+k_{con}+k_{rad}$$

where $k_{sol}$ and $k_{gas}$ are the conduction through the solid and air, respectively, $k_{con}$ is the convection within the cells, and $k_{rad}$ is the radiation through the cell walls and across the cell voids [33].

The largest portion that would compose the difference of thermal conductivity values among samples would be the conduction through air,$\;k_{gas}$ (material with open pores), as it is the same polymer for all samples, and $k_{sol}$ is the same for all samples. The conduction by convection within cells ($k_{con}$) is negligible, and this portion is only considered for pores larger than 10 mm [33]. The radiative portion would not influence the difference among samples since the materials have low densities, and the tests were performed at same temperatures (directly proportional to T^3^). Among all samples, AOT7.25 presented the smallest lowest *k* value because it contains the largest surface area (A_sp_) and the smallest pore size (Table 3), which results in it yielding the largest pore volume. On the hand, K2.75 and K5 presented irregular large pores, as evidenced in Figure 3c,d, respectively, resulting in low pore volume and high *k* values. 

**Figure 6:**
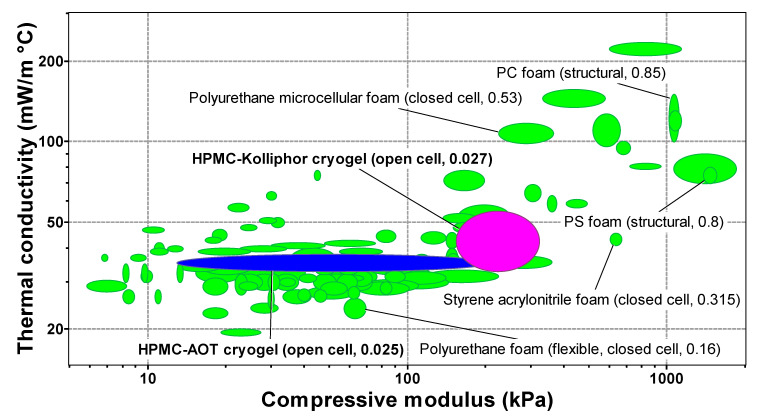
Ashby chart of thermal conductivity vs. compressive modulus obtained by the software CES Edupack 2019^®^ at a level 3 showing 106 classes of materials belonging to the family of foams. The numbers in the parenthesis correspond to the relative density, which is the apparent density divided by the density of bulk material; the density of bulk HPMC amounts to 1326 kg/m^3^. The blue and magenta regions were introduced in the chart to show the position of the cryogels prepared with hydrogel precursors containing Kolliphor^®^ EL and AOT, respectively.

Figure 6 shows the Ashby chart of thermal conductivity vs. compressive modulus obtained by the software CES Edupack 2019^®^ at a level 3 showing 106 classes of materials belonging to the family of polymeric foams. The Young modulus and thermal conductivity values obtained for the cryogels investigated in this study were inserted in the Ashby diagram in order to compare them with other types of polymeric foams. Cryogels prepared with AOT occupy the lower left position (blue); the compressive modulus can be tuned by the amount of AOT added to the precursor hydrogel, keeping low thermal conductivity. Cryogels prepared with Kolliphor^®^ EL occupied a region (magenta) characteristic of higher compressive modulus and thermal conductivity values; the thermal conductivity can be tuned by the content of Kolliphor^®^ EL in the precursor hydrogel.

The thermal stability of the cryogels and the pure HPMC, Kolliphor^®^ EL, and AOT was evaluated using TG/DTG curves (Figure S4). The maximum thermal decomposition temperatures ($T_{max}$) of K0.5, K7.25, and AOT0.5 were slightly lower than those of pure the HPMC and Kolliphor^®^ EL (Table 4). Nevertheless, the $T_{max}$ value of AOT7.25 was similar to that of the HPMC cryogels, and the residual mass at 500 °C of AOT7.25 was higher (11.78%) than that observed for HPMC (0.47%), indicating favorable interactions between HPMC and AOT when the AOT concentration in the precursor was the highest. 

The difference of mass (Δ%m) between 30 °C ($\%m_{30{}^{\circ}C}$) and 100 °C ($\%m_{100{}^{\circ}C}$) was attributed to water loss (Figure S5). The cryogels lost between 5.7% and 7.2% of their water, except for AOT7.25, which only lost 0.6%. This low amount of water loss indicates that the cell walls of AOT7.25 are less hygroscopic than those in the other cryogels are. Hygroscopicity is a common issue with polysaccharide-based cryogels because it affects the thermal conductivity [26]. 

The swelling degrees (SD) of the HPMC cryogels, K0.5, AOT 0.5 (lowest surfactant concentration), and K7.25 (highest surfactant concentration) were similar, ranging from 32 ± 1 g_water_/g to 29 ± 1 g_water_/g (Figure S6). However, AOT7.25 (the highest surfactant concentration) presented a SD of 22 ± 1 g_water_/g, indicating less affinity to water and corroborating the low amount of water loss in the temperature range from 30 °C to 100 °C.

**Figure 7:**
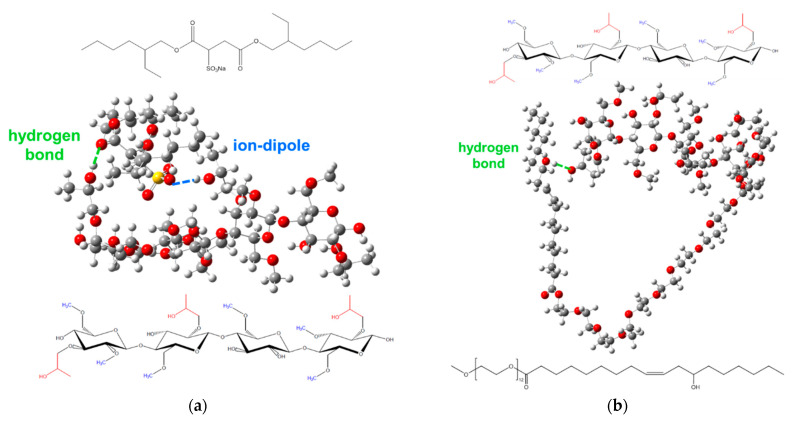
Optimized geometries obtained from DFT simulation in vacuum for (**a**) HPMC–AOT, where the AOT sulfonate group approaches the HPMC hydroxyl group through ion–dipole interaction and through the hydrogen bond between the AOT carbonyl group and the HPMC hydroxyl group and (**b**) the HPMC-Kolliphor^®^ EL fragment, where the Kolliphor^®^ EL hydroxyl group interacts with the HPMC hydroxyl group through the hydrogen bond.

Density Functional Theory (DFT) calculations were performed in order to gain insight into the molecular interactions between the surfactants and the HPMC in vacuum, which might be correlated to the molecular interactions in the cryogels. The optimized geometries indicated an interaction energy of −31.8 kcal/mol for the HPMC–AOT system, driven by the strong interaction between the AOT sulfonate group (ion) and the HPMC hydroxyl group (dipole) and the hydrogen bond between the AOT carbonyl group and the HPMC hydroxyl group, as depicted in Figure 7a. Favorable interactions (−37.93 kcal/mol) were also predicted by DFT calculations for the AOT–water–ethylene glycol system [34]. In the case of the HPMC–Kolliphor^®^ EL system, the calculated interaction energy was −7.91 kcal/mol. Figure 7b shows the optimized geometry obtained from the DFT simulation for HPMC–Kolliphor^®^ EL, where only one H bond between the Kolliphor^®^ EL hydroxyl and the HPMC hydroxyl groups drives the interaction.

**Figure 8:**
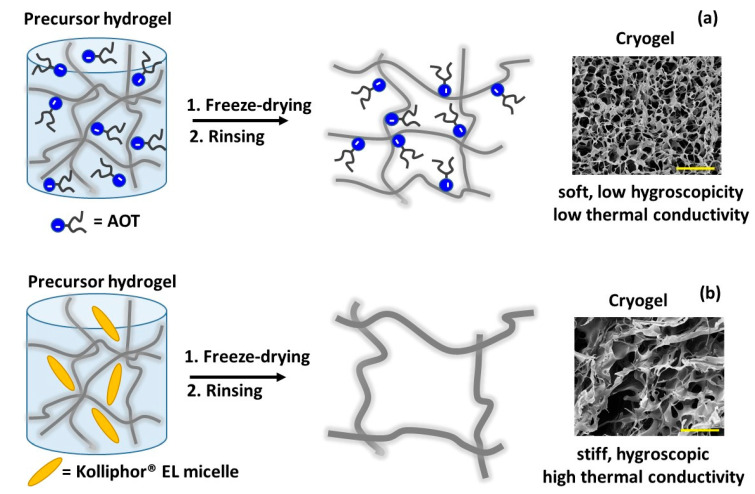
Schematic representation of the model proposed to explain the effects of adding (**a**) AOT or (**b**) Kolliphor^®^ EL to the precursor hydrogel on the microstructure and the properties of the resulting cryogels. After rinsing to remove the molecules that did not react, the cryogels were freeze-dried again and were characterized. Due to the strong interactions between the AOT and HPMC, the AOT molecules remained adsorbed on the HPMC cell walls, rendering softness and less hygroscopic cryogels. The Kolliphor^®^ EL molecules promoted the formation of larger ice crystals and consequently larger pores and thicker cell walls.

The relation between the microstructure and the mechanical/thermal properties of HPMC cryogels could be tuned by the concentration of AOT added to the precursor HPMC hydrogels; the highest AOT concentrations (AOT5 or AOT7.25) led to soft cryogels with a relatively low thermal conductivity, which were associated to a microstructure of many small pores and thin cell walls. Furthermore, these cryogels were less hydrophilic than the control (HPMC cryogel). AOT is frequently used as an emulsifier in water/oil emulsion, building reverse micelles, where the aggregation number is usually low (*n* < 20) [35]. In HPMC hydrogels there is no oil phase, and a loose packing is expected [19]. Based on the DFT calculations and the experimental observations, a model is proposed to explain the microstructure formation. In the hydrogels containing AOT (at concentration above the cmc), the AOT sulfonate and carbonyl groups are orientated to the HPMC chains. The loose packing affects the hydration of the hydrophobic alkyl portions, and the surrounding water molecules would be arranged as small clusters. The distribution of water into many small clusters would promote a more homogeneous distribution of the HPMC chains in the matrix. Upon freezing, the small clusters of liquid water would crystallize into smaller ice crystals surrounded by thin HPMC walls. The sublimation of small ice crystals during the freeze-drying process would create small pores in the cryogels. After crosslinking, rinsing to remove the unreacted molecules and then freeze-drying again, a monolayer of AOT with the double chains oriented to the air would remain on the HOMC cell walls, rendering lubricity and softness for the cryogels. Cationic softeners for fibers (textile or hairs) behave in a similar way. They are cationic surfactants that adsorb on the negatively charged surface and orient the hydrophobic chains to the air, bringing lubricity and softness to the fibers [36]. Therefore, the presence of AOT molecules in the hydrogels would lead to smaller pores (smaller ice crystals) and less hygroscopic cell walls, compared to pure HPMC cryogels, as depicted in Figure 8a. If the AOT concentration in the hydrogel is low (AOT0.5 and AOT2.75), the amount of water arranged in small clusters would be not so pronounced, causing less of an effect on the ice crystallization, and the resulting cryogels would be similar to those prepared without AOT.

The concentration of Kolliphor^®^ EL added to the precursor hydrogels led to cryogels with a high compressive modulus and high thermal conductivity compared to HPMC cryogels. DFT calculations evidenced that the interactions between Kolliphor^®^ EL and HPMC are not as strong as those between AOT and HPMC are. The presence of the Kollipho^®^ EL micelles [30] possibly led to an increase in the size of the ice crystals, compressing the HPMC chains. This effect would lead to thicker cell walls and larger pores, causing an increase in the compression modulus and the thermal conductivity, as depicted in Figure 8b.

## 3. Conclusions

This study demonstrated that it is possible to tune the microstructure of the cryogel and consequently their properties by adding surfactant to the precursor hydrogels. Particularly, the favorable interactions between the AOT polar head and the HPMC chains in the precursor hydrogel led to soft (E = 13 kPa) cryogels with relatively low thermal conductivity (0.033 W m^−1^ °C^−1^) and low hygroscopicity. The interactions between Kolliphor^®^ EL and HPMC are weak; the Kolliphor^®^ EL micelles probably increased the ice crystal size, yielding stiffer cryogels. The stiffest cryogels (E = 55 kPa) presented the highest thermal conductivity value (0.055 W m^−1^ °C^−1^). By changing the type and concentration of the surfactant in the precursor hydrogels, a wide range of compressive modulus and thermal conductivity values could be achieved (Figure 6). Therefore, it is a simple strategy to create cellulose-based cryogels with tunable properties to replace foams made of synthetic polymers.

## 4. Materials and Methods

### 4.1. Materials

**Figure 9:**
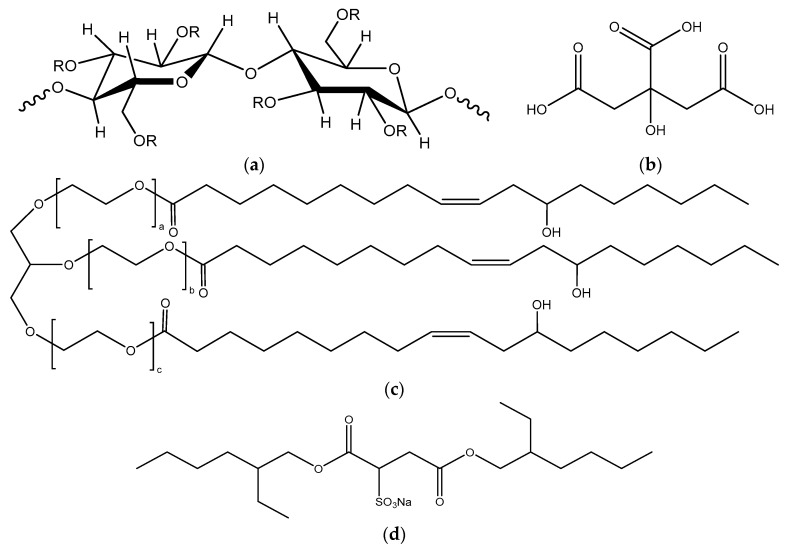
Schematic representation of chemical structures of (**a**) HPMC, with R = -H, -CH_3_, or -(CH_2_-CHOH-CH_3_)_n_; (**b**) citric acid; (**c**) Kolliphor^®^ EL, where a + b + c = 35 [30]; and (**d**) AOT.

Commercial HPMC J12MS with a degree of substitution (DS) of 1.5 and a molar substitution (MS) of 0.75 (USP 1828) was kindly supplied by The Dow Chemical Company (Sao Paulo, Brazil); the viscometric average molar mass ($M_{v}$) of 3.46 × 10^5^ g mol^−1^ was determined through capillary viscometry (Figure S7). Kolliphor^®^ EL (Polyoxyl-35 castor oil, C5135 Sigma-Aldrich, Sao Paulo, Brazil, ~2500 g/mol), dioctyl sulfosuccinate salt sodium or AOT (323586 Sigma-Aldrich, Sao Paulo, Brazil, 444.56 g/mol), citric acid (Labsynth, Sao Paulo, Brazil, 192.12 g mol^−1^), and sodium hypophosphite (Labsynth, Sao Paulo, Brazil, 87.98 g mol^−1^) were used as received. MilliQ water was employed in all experiments. The critical micelle concentration (cmc) of AOT and Kolliphor^®^ EL in water were determined as 1.9 mM and 0.02 mM, respectively, at 21 ± 1 °C (Figure S8a,b); these values are in agreement with literature data [37,38]. Figure 9 represents the chemical structures of HPMC, citric acid, Kolliphor^®^ EL, and AOT.

### 4.2. Preparation of HPMC Cryogels

Aqueous solutions of AOT and Kolliphor^®^ EL were prepared at 0.2, 1.0, 2.0, and 3.0 mM under magnetic stirring at 23 ± 1 °C. Citric acid (crosslinker) and sodium hypophosphite (catalyst) were then added at 1.0 g·L^−1^ and 0.5 g·L^−1^; these concentrations were chosen based on previous reports [20]. After 30 min of stirring, HPMC was added to the aqueous solutions at the final concentration of 30 g·L^−1^ and was homogenized for 1 h. The resulting hydrogels were coded as HPMC (blank, without surfactant), K0.5 and AOT0.5 (0.2 mM), K2.75 and AOT2.75 (1.0 mM), K5 and AOT5 (2.0 mM), and K7.25 and AOT7.25 (3.0 mM), as shown in Table 1. One should notice that the concentration of Kolliphor^®^ EL in all gels was above of its cmc (0.02 m)M, whereas the concentration of AOT in the AOT0.5 and AOT2.75 gels was below its cmc, and in the AOT5 and AOT7.25 gels, it was above its cmc. 

**Figure 10:**
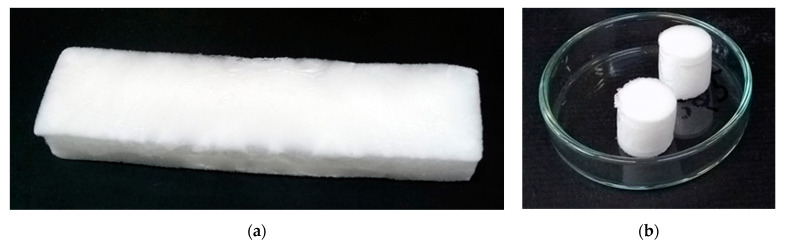
Photographs of freeze-dried (**a**) AOT7.25 (obtained by mold A) and (**b**) K7.25 (mold B) samples.

The precursor hydrogels were transferred to acrylic molds of different geometries: A (rectangular, sample size: 28 mm × 115 mm × 15 mm), B (cylindrical, ø = 14 mm × 15 mm), C (cylindrical, ø = 14 mm × 10 mm), D (cylindrical, ø = 10 mm × 6 mm), and E (cylindrical, ø = 5 mm × 5 mm). The molds were covered in order to avoid significant volume variation due to ice crystallization during freezing. They were kept inside three Styrofoam boxes (Figure S9) for 24 h in a standard freezer at −33 °C in order to promote isotropic cooling [39]. They were then freeze-dried under vacuum (~0.2 mbar) for 36 h (mold A) or 12 h (molds B–E). After freeze-drying, the cryogels were heated for 7 min at 165 °C in order to promote crosslinking (esterification) between the citric acid and the hydroxyl groups from the HPMC chains [20]. Figure 10 shows photographs of typical cryogels prepared in molds A and B.

### 4.3. Characterization of Precursor Hydrogels

Dynamic strain sweep tests (DSST), dynamic time sweep tests (DTST), and small amplitude oscillatory shear (SAOS) tests were performed for the hydrated samples using a stress-controlled MCR 501^®^ rheometer from Anton Paar at 25.0 ± 0.5 °C under a dry nitrogen atmosphere. A cone and plate geometry (angle of 0.992°) with a gap size of 0.101 mm and a diameter of 50 mm was used. For DSST, pure HPMC (blank), K7.25, and AOT7.25 hydrogels were tested at the frequencies of 1 Hz and 10 Hz, measuring G′, G″ as a function of the strain amplitude (Figure S10). In order to evaluate the storage (G′) and loss (G″) moduli and the damping factor (tan δ = G″/G′) of the precursor hydrogels, SAOS tests were performed by varying the frequency from 0.1 to 10 Hz, keeping the strain at 0.5% (Figure S11). The obtained value of 0.5% strain was adopted for all samples in the subsequent tests since for this strain, the response of the samples was within the linear viscoelastic region. The HPMC, K7.25, and AOT7.25 hydrogels showed high stability over 900 s at a frequency of 1 Hz and a strain of 0.5. It should be pointed out that all of the rheological measurements were repeated up to 3 times, and the data were observed to be reproducible within ±5%.

### 4.4. Characterization of the Cryogels

Prior to the characterization, all of the cryogels were rinsed with MilliQ water (~100 mL) until the rinsing water achieved the conductivity of ~5 µS·cm^−1^ in order to remove unreacted molecules. The cryogels were then frozen and freeze-dried again. The apparent density (*ρ_ap_*) of six dried cryogels (mold B) was determined at 23 ± 1 °C and relative air humidity of 65 ± 10%. The ratio between their masses ($m_{pol}$) was obtained using an analytical balance, and their volumes measured by a caliper.

To determine the gel content (percentage of crosslinked HPMC chains), the cryogels (mold C) were swollen with MilliQ water, freeze-dried, and weighed ($m_{dried}$). The gel content (% Gel) was calculated using Equation (2):

(2) $$\%Gel=\frac{m_{dried}}{m_{pol}}\times 100\%$$

The swelling degree (SD) was determined for HPMC, K0.5, AOT0.5, K7.25, and AOT7.25 (mold C) samples using a force tensiometer Krüss K100 at 21.0 ± 0.5 °C as the mass of sorbed MilliQ water (pH 5.5) at equilibrium (*m_water_*) divided by *m_dried_*, as shown by Equation (3):

(3) $$SD=\frac{m_{water}}{m_{dried}}$$

FTIR-ATR vibrational spectra of the HPMC, K0.5, AOT0.5, K7.25, and AOT7.25 samples (mold C) were obtained using an Alpha FTIR-ATR spectrometer (Bruker^®^) with a diamond crystal and an accumulation of 128 scans (2 cm^−1^ resolution). Thermogravimetric analyses (TGA) were performed for the HPMC, K0.5, AOT0.5, K7.25, and AOT7.25 samples (mold C) in alumina crucibles using the Netzsch STA 449 F3 Jupiter (Netzsch^®^) equipment under a N_2_ atmosphere. The temperature program had three segments: 1 (from 30 °C to 200 °C at a heating rate of 20 °C/min), 2 (from 200 °C to 30 °C at a cooling rate of −20 °C/min), and 3 (from 30 °C to 500 °C at a heating rate of 10 °C/min). The first run up to 200 °C or well below the degradation point was performed in order to remove thermal history and moisture. Afterwards, the second run was performed slowly at the heating rate of 10 °C/min to observe the actual thermal stability in TGA and its char yield.

The thermal conductivity, k, of the cryogels (mold A) was measured using the Transient Hot Strip (THS) method [32,39,40,41]. In this method, a constant linear electric power ($P_{0}$) is supplied to a resistive strip, which heats the sample. The temperature variation between the sample (near the strip) and the external environment (ΔT) is measured as function of time (t). The slope, m, of the linear region of the dependence of ΔT on ln t is used to calculate the k value:

(4) $$k=f\frac{P_{0}}{4\pi .m}$$

where f is the correction factor obtained by measuring an open cell polyurethane (PU) foam (“reference”, k = 25 mW m^−1^ °C^−1^, $\rho_{ap}$ = 13.5 kg/m^3^ [42]). The correction factor f was 0.03546 obtained after measuring the PU reference.

**Figure 11:**
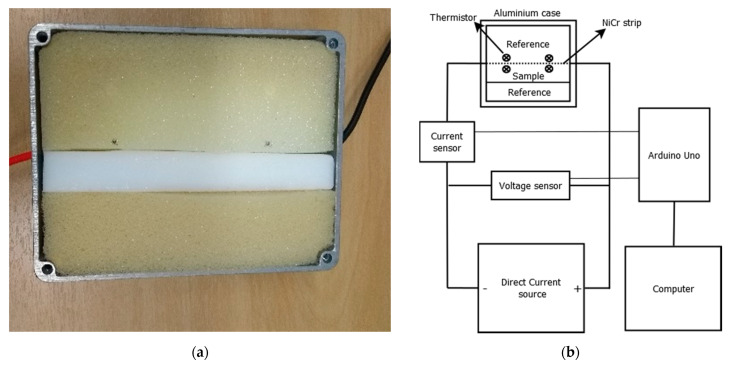
(**a**) Top-view of the aluminum case (open) filled with the sample and the reference (PU) and (**b**) the schematic representation of the experimental setup. There were four thermistors that were also connected to the Arduino Uno board. The dotted line represents the NiCr strip.

All measurements of thermal conductivity were performed with a low cost (~USD 100) homemade device. It comprises an isothermal aluminum case with a NiCr resistive strip (2.0 mm × 0.1 mm × 110.0 mm) positioned at the center of the case, and one of the sample faces was against the strip. One piece of PU foam (reference) was placed against the other side of the strip, and another piece of PU foam was placed against the sample, as shown in Figure 11a. There were two thermistors that were inserted through the case cover and were passed through the sample next to the strip in order to obtain an average sample temperature over time. There were two other thermistors that were only inserted through the case cover to measure its average temperature over time. The system was connected to a DC source and two sensors (current and voltage) calibrated with a multimeter EDA MAS838L^®^ in order to obtain the electric power supplied to the strip over time. An Arduino Uno R3 board was used to transfer the acquired data to the computer (Figure 11b). The tests were performed in triplicate for 7 min at 23.7 ± 0.7 °C and at 60% relative humidity. There were three types of power (1.0, 2.0 and 3.0 W) that were supplied by the direct current source, and an average value was taken from the obtained thermal conductivities. From the k value of the PU foam, the values obtained from the k of the samples could be corrected. A one-way analysis of variance (ANOVA) with a post hoc test was used to evaluate the differences among the groups. Results of *p* < 0.05 were considered statistically different. Analyses were performed with Excel 2013^®^ for Windows^®^ (Microsoft Office Home and Student^®^, 2013).

The compressive tests were performed in quadruplicate for the cryogel samples (mold B) using an Impac Digital Dynamometer IP-90DI with a 1 kN load cell at strain rate of 0.01 s^−1^ and at an air temperature of 23 ± 1 °C (60% relative humidity). The samples remained close to the dynamometer under 60% relative humidity for 24 h as recommended by ISO 3386-1 [43]. 

Freeze-dried cryogels (mold D) coated with a gold layer (~10 nm) were analyzed in a JEOL Neoscope JCM-5000 microscope operating at voltage of 10 kV. The cryogels (mold E) were analyzed using X-ray microtomography (micro-CT) with Bruker Skyscan 1272 equipment with the X-ray source operating at 20 kV and a current of 175 μA. The samples were rotated 0.6° per step, obtaining a resolution of 4 μm. The NRecon (v. 1.6.9.8, SkyScan) and CTVOx (v. 2.2.3.0, SkyScan) software programs were used for the 3D reconstruction from cross-sectional images and for the visualization and image acquisition, respectively. Specific surface area values of volumes of interest (VOI) were calculated using the software CTan (v. 1.14.4.1, SkyScan). The analyses of the VOIs with the CTan^®^ software were the specific area $(A_{sp}$), calculated by Equation (5); connectivity density (the average number of connections in 1 mm^3^); and average pore size. The pore size distributions in the HPMC, K7.25, and AOT7.25 samples were calculated with CTan^®^ software.

(5) $$A_{sp}\;=\;\frac{A}{\rho .VOI}$$

where *VOI* (volume of interest) is the reconstructed volume from which the software performed the calculations, A is the internal area of the *VOI*, and *ρ* is the material density, which was considered as 34 kg/m^3^ (Table 2).

**Figure 12:**
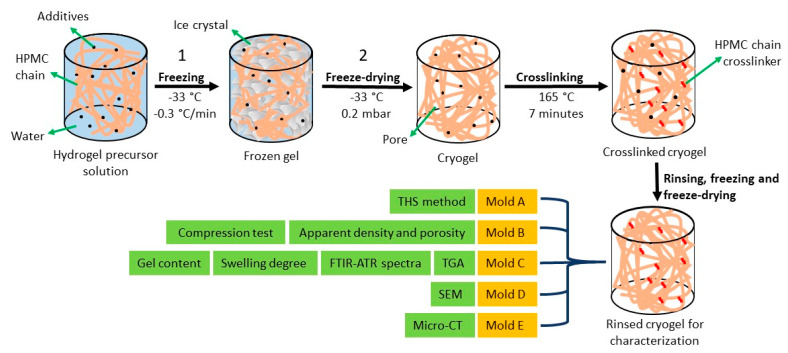
Experimental procedure for the cryogel preparation and characterization. The samples were molded in five different molds (A–E) of different sizes and shapes. Each type of analysis required samples prepared with a specific mold due to the size of rgw sample holder or the intrinsic measurement condition. The additives represent Kolliphor^®^ EL, AOT, citric acid, and sodium hypophosphite.

Figure 12 shows the experimental procedure for the cryogels preparation and characterization schematically.

### 4.5. Density Functional Theory (DFT) Calculations

Density Functional Theory (DFT) calculations were performed using the software Gaussian 09 [44] with the RB3LYP functional and the 6-31G(d) as the base, and to account for the basis set superposition error (BSSE), the counterpoise correction was applied to evaluate the interaction energies between AOT–HPMC and Kolliphor^®^ EL–HPMC in vacuum. For the calculations, an HPMC tetramer containing 6 -CH_3_, 3 -CH_2_-CHOH-CH_3_, and 5 OH groups and one third of the Kolliphor^®^ EL molecule were drawn (the -O-CH_2_- bond from the precursor glycerol was considered as -O-CH_3_), as shown in Figure 9a,b, respectively. To model the interactions, only the fragments were considered due to the high computational cost.

## Figures and Tables

**Table 1:** Hydrogel composition and the corresponding codes. All hydrogels contained HPMC at 30 g·L^−1^, citric acid at 1.0 g·L^−1^ and sodium hypophosphate at 0.5 g·L^−1^. The concentrations of Kolliphor^®^ EL and AOT varied from 0.2 mM to 3.0 mM.

Code	HPMC	K0.5	AOT0.5	K2.75	AOT2.75	K5	AOT5	K7.25	AOT7.25
[Kolliphor^®^ EL] mM	-	0.2	-	1.0	-	2.0	-	3.0	-
[AOT] mM	-	-	0.2	-	1.0	-	2.0	-	3.0

**Table 2:** Mean values (*n* > 4) of apparent density (*ρ_ap_*), gel content (Gel%) in MilliQ water, Young’s moduli (E), and yield stress ($\sigma_{y}$) obtained for all cryogels. tan *δ* (G″/G′) determined by SAOS at 0.1 Hz for the precursor hydrogels.

Sample	*ρ_ap_* (kg/m^3^)	Gel (%)	E (kPa)	$\sigma_{y}$ (kPa)	tan *δ*
HPMC	34 ± 3	86 ± 1	154 ± 15	7.7 ± 0.8	2.89 ± 0.09
K0.5	33 ± 2	87 ± 2	320 ± 32	16 ± 2	2.91 ± 0.09
K2.75	35 ± 3	67 ± 2	202 ± 20	10 ± 1	3.16 ± 0.09
K5	37 ± 3	63 ± 2	234 ± 23	12 ± 1	2.96 ± 0.09
K7.25	39 ± 5	61 ± 1	283 ± 28	14 ± 1	2.73 ± 0.08
AOT0.5	32 ± 4	88 ± 3	205 ± 20	10 ± 1	2.89 ± 0.09
AOT2.75	34 ± 3	86 ± 2	176 ± 18	8.8 ± 0.9	3.18 ± 0.09
AOT5	31 ± 4	86 ± 1	39 ± 4	2.0 ± 0.2	3.03 ± 0.09
AOT7.25	34 ± 2	84 ± 1	13 ± 1	0.65 ± 0.07	2.91 ± 0.09

**Table 3:** Volume of interest (VOI), internal area of the VOI (*A*), connectivity density (the average number of connections in 1 mm^3^, specific area $(A_{sp}$)), and mean pore size calculated with CTAn^®^ software.

Sample	VOI (mm^3^)	A (mm^2^)	Connectivity Density (mm^−3^)	$A_{sp}$ (m^2^/kg)	Mean Pore Size (μm)
HPMC	1.302	82.2	25,482	1856	34.0 ± 10.0
K7.25	1.322	49.9	8570	1110	45.8 ± 9.0
AOT7.25	1.273	99.9	29,900	2308	25.3 ± 9.4

**Table 4:** Maximum thermal decomposition temperature ($T_{max}$), percentage in residual mass at 500 °C ($\%m_{500{}^{\circ}C}$), and difference of mass (Δ%m) between 30 °C ($\%m_{30{}^{\circ}C}$) and 100 °C ($\%m_{100{}^{\circ}C}$) determined for HPMC (powder), pure Kolliphor, pure AOT, HPMC, K0.5, K7.25, AOT0.5, and AOT7.25 cryogels.

Sample	$T_{max}$ (°C)	$\%m_{500{}^{\circ}C}$	Δ%m
HPMC (powder)	363	9.52	4.9
Kolliphor (pure)	415	1.84	2.5
AOT (pure)	293	15.53	3.8
HPMC	358	0.47	5.7
K0.5	340	6.22	6.7
K7.25	344	3.90	6.2
AOT0.5	327	5.93	7.2
AOT7.25	356	11.78	0.6

## Supplementary Materials

The following are available online at https://www.mdpi.com/article/10.3390/gels7030118/s1, Figure S1: FTIR-ATR spectra. Figure S2: Histogram of average pore size. Figure S3: ΔT vs. ln t. Figure S4: TG/DTG curves. Figure S5: TG curves. Figure S6: Water sorption and swelling degree experiments. Figure S7: Determination of viscometric average molar mass of HPMC. Figure S8: Surface tension vs. logarithm of the concentration (mM) of (a) AOT and (b) Kolliphor^®^ EL. Figure S9: (a) Styrofoam boxes arranged for the precursor gels freezing; (b) top-view representation of boxes (gray) with the distances in millimeters among the molds (symmetrically positioned in the center) and boxes as well as their wall thickness and size. (c) The temperature of points A (hydrogel sample in mold A), B, C, and D (outer surface of each box) was measured using thermistors as a function of time. Figure S10: Storage (G′) and loss (G″) moduli obtained by DSST tests for the hydrogels. Figure S11: Curves obtained by the SAOS tests performed at 25.0 ± 0.5 °C.
