# Supplementary material for: Tuning the Mechanical and Thermal Properties of Hydroxypropyl Methylcellulose Cryogels with the Aid of Surfactants
Source: Gels. 2021 Aug 11;7(3):118. doi: 10.3390/gels7030118 (PMC8396048; doi:10.3390/gels7030118)
Supplement: Supplementary file 1 [file gels-07-00118-s001.zip › gels-1326085-supplementary.pdf]

Supplementary Material

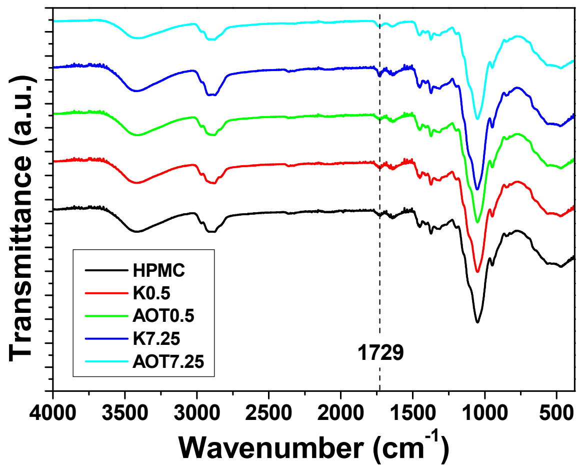

(a)

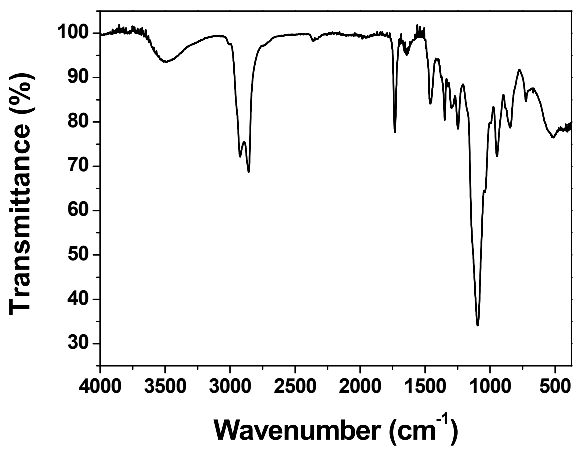

pure Kolliphor® EL (b)

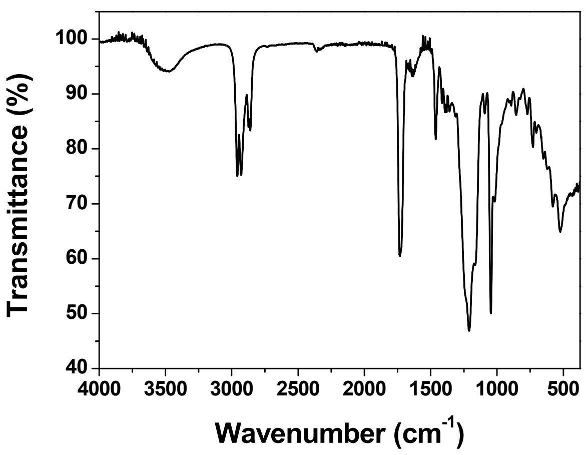

pure AOT (c)

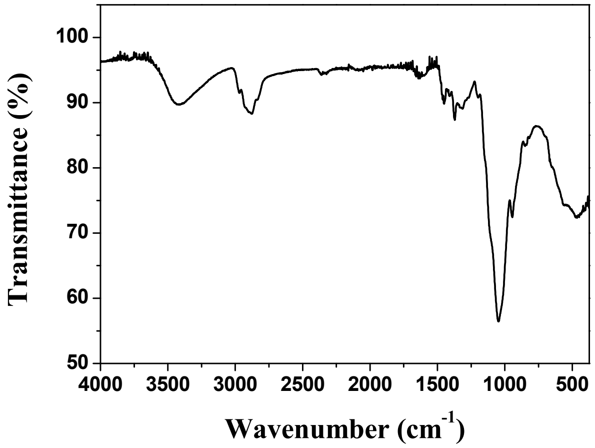

pure HPMC **(d)**

Band (cm^{-1})	Assignment	Reference
3419	Stretching vibration of bonded and non-bonded –OH groups	[21]
2921<br>2880	Asymmetric $–CH_2–$, symmetric $–CH_3$ and $–CH_2–$ stretching vibrations	
1729	Ester –C=O stretching vibrations	
1633	Vibration of water molecules absorbed in HPMC	[22]
1460<br>1370<br>1053<br>947	Stretching and bending vibrations of -CH2 and -CH, -OH and C-O bonds in glycosidic ring	

(e)

Figure S1. FTIR-ATR spectra (128 scans, 2 cm^{-1} resolution) of (a) HPMC, K0.5, K7.25, AOT0.5 and AOT7.25 cryogels, (b) pure Kolliphor® EL, (c) pure AOT, (d) pure HPMC and (e) band assignments.

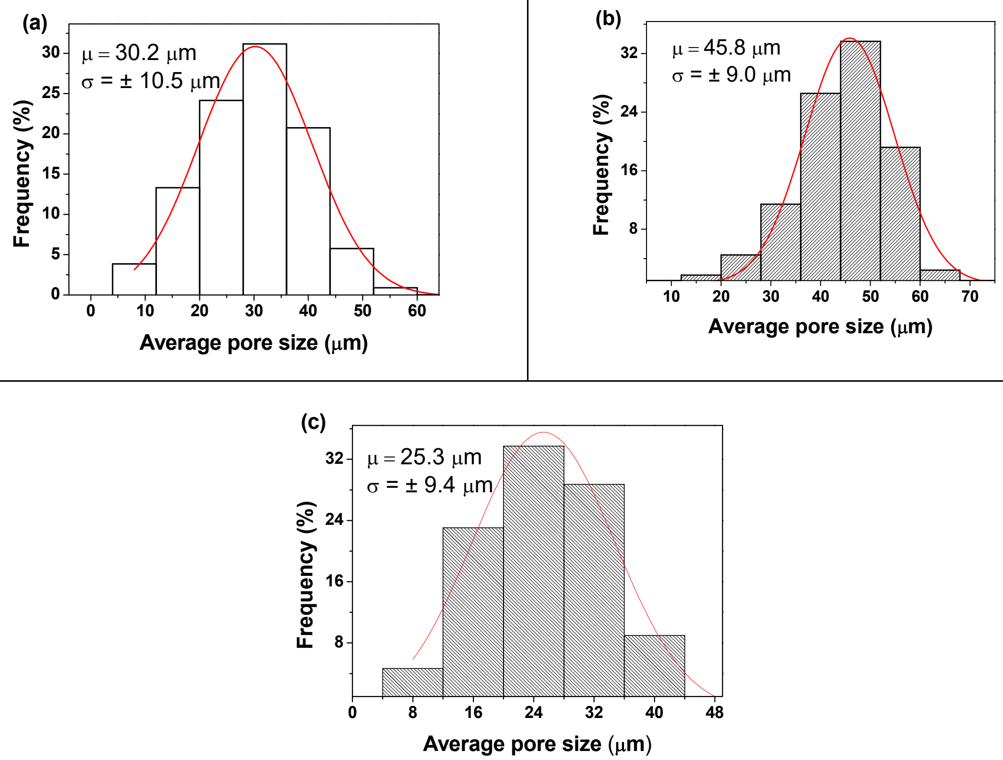

Figure S2. Histograms of average pore size calculated by the software CTan® for (A) HPMC, (B) K7.25 and (C) AOT7.25 cryogel samples.

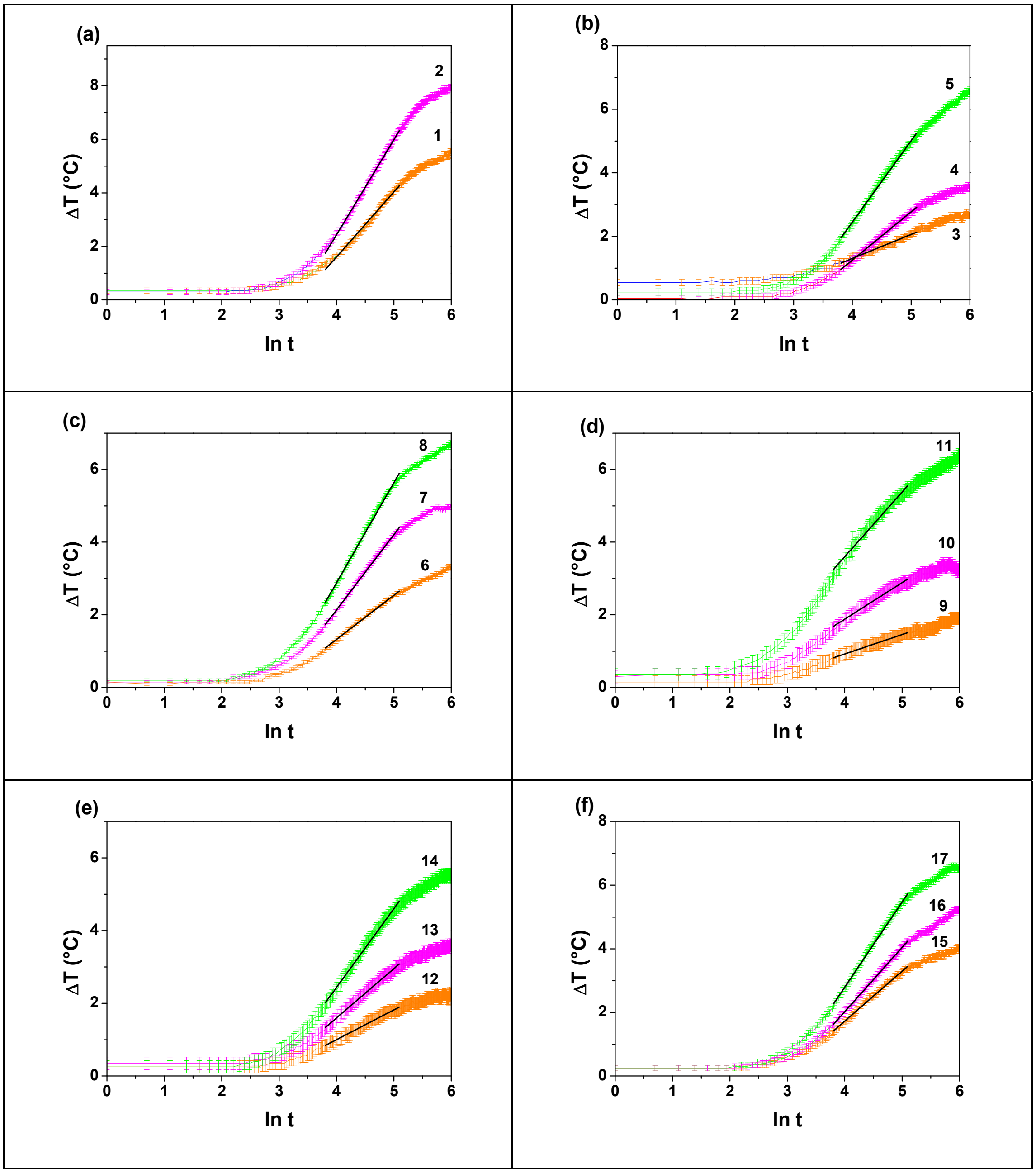

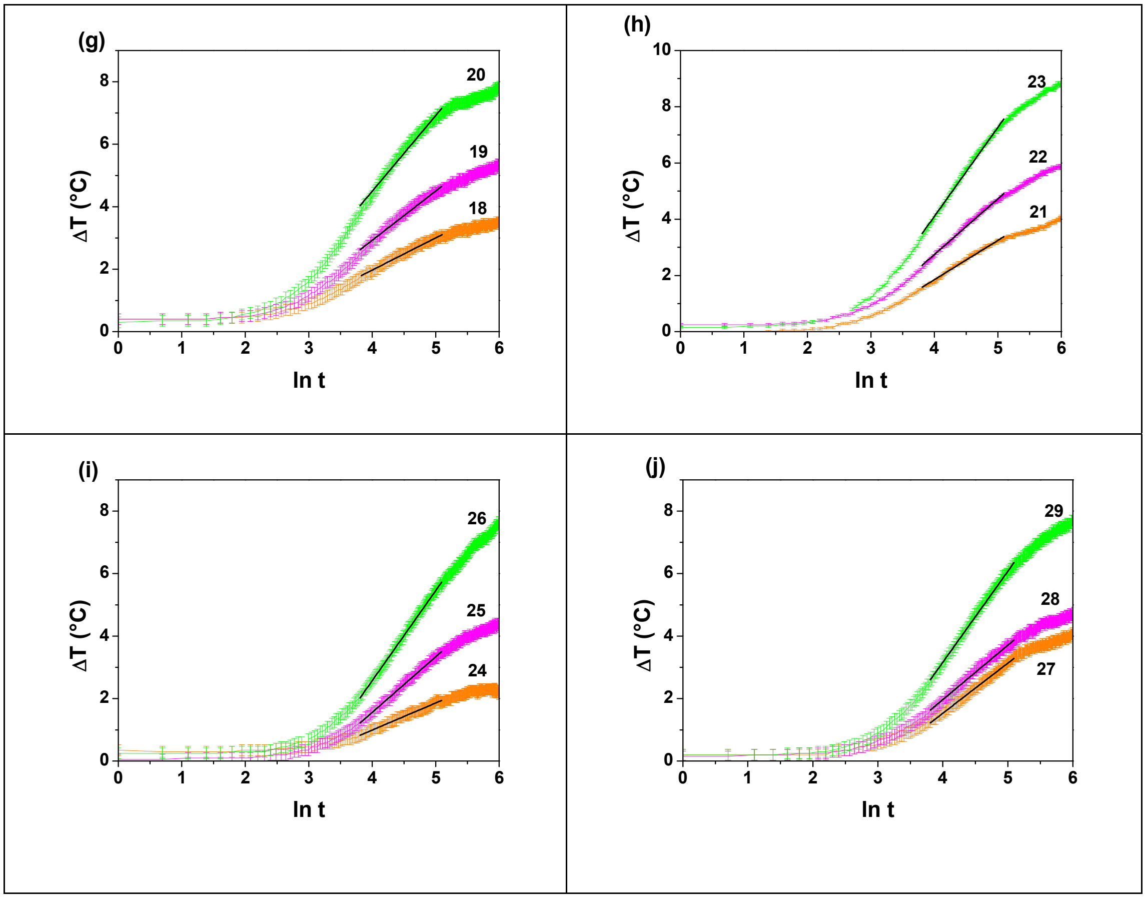

Curve	Electric power supplied P (W)	Linear region			Uncorrected thermal conductivity k ($W.m^{-1}.°C^{-1}$)
		Slope	Intercept	R^2	
1	2.17 ± 0.04	2.43 ± 0.02	-11.8 ± 0.1	0.99343	0.071 ± 0.002
2	3.11 ± 0.05	3.55 ± 0.02	-8.1 ± 0.1	0.99843	0.070 ± 0.001
3	1.16 ± 0.03	0.75 ± 0.02	-1.7 ± 0.1	0.98026	0.123 ± 0.004
4	2.84 ± 0.06	1.52 ± 0.02	-4.8 ± 0.1	0.99622	0.093 ± 0.002
5	3.22 ± 0.07	2.56 ± 0.02	-7.8 ± 0.1	0.99577	0.100 ± 0.002
6	1.48 ± 0.02	1.21 ± 0.01	-3.53 ± 0.05	0.99387	0.177 ± 0.017
7	2.28 ± 0.02	2.07 ± 0.01	-6.14 ± 0.05	0.99653	0.177 ± 0.016
8	3.06 ± 0.02	2.76 ± 0.01	-8.14 ± 0.05	0.99536	0.177 ± 0.015
9	1.38 ± 0.04	0.53 ± 0.04	-1.2 ± 0.2	0.98576	0.205 ± 0.006
10	1.81 ± 0.03	1.00 ± 0.04	-2.1 ± 0.2	0.98360	0.143 ± 0.002
11	3.12 ± 0.04	1.78 ± 0.04	-3.5 ± 0.2	0.98204	0.139 ± 0.002
12	1.45 ± 0.06	0.82 ± 0.04	-2.3 ± 0.2	0.98578	0.118 ± 0.005
13	2.06 ± 0.01	1.35 ± 0.04	-3.8 ± 0.2	0.99489	0.121 ± 0.007
14	2.90 ± 0.02	2.17 ± 0.04	-6.2 ± 0.2	0.99356	0.106 ± 0.001
15	1.90 ± 0.02	1.57 ± 0.01	-4.56 ± 0.05	0.99332	0.096 ± 0.001
16	2.10 ± 0.02	2.02 ± 0.01	-6.07 ± 0.04	0.99814	0.090 ± 0.001
17	3.03 ± 0.04	2.68 ± 0.01	-2.68 ± 0.04	0.99753	0.083 ± 0.001

18	1.49 ± 0.01	1.03 ± 0.01	-2.14 ± 0.05	0.98796	0.114 ± 0.004
19	2.25 ± 0.02	1.57 ± 0.02	-3.38 ± 0.08	0.98744	0.110 ± 0.003
20	3.25 ± 0.03	2.42 ± 0.03	-5.2 ± 0.1	0.98573	0.104 ± 0.002
21	1.70 ± 0.05	1.39 ± 0.01	-3.74 ± 0.05	0.99002	0.097 ± 0.003
22	2.14 ± 0.02	1.98 ± 0.01	-5.17 ± 0.05	0.99356	0.086 ± 0.001
23	1.97 ± 0.03	3.16 ± 0.01	-8.56 ± 0.05	0.99496	0.092 ± 0.002
24	1.07 ± 0.01	0.87 ± 0.04	-2.5 ± 0.2	0.98797	0.100 ± 0.001
25	1.97 ± 0.02	1.78 ± 0.04	-5.6 ± 0.2	0.99722	0.092 ± 0.002
26	3.11 ± 0.01	2.88 ± 0.04	-9.0 ± 0.2	0.99861	0.093 ± 0.001
27	1.11 ± 0.01	1.60 ± 0.04	-4.9 ± 0.2	0.99743	0.177 ± 0.004
28	1.92 ± 0.01	1.73 ± 0.04	-5.0 ± 0.2	0.99389	0.109 ± 0.001
29	2.98 ± 0.02	2.91 ± 0.04	-8.5 ± 0.2	0.99712	0.097 ± 0.001

Figure S3. ΔT vs ln t obtained at 23.7 ± 0.7 °C for (a) reference (PU); (b) HPMC; (c) K0.5; (d) K2.75; (e) K5; (f) K7.25; (g) AOT0.5; (h) AOT 2.75; (i) AOT5; (j) AOT7.25. *P* is the measured supplied electric power and *k* is the calculated thermal conductivity. The linear region considered was between 46 (ln t = 3.83) and 164 (ln t = 5.10) s.

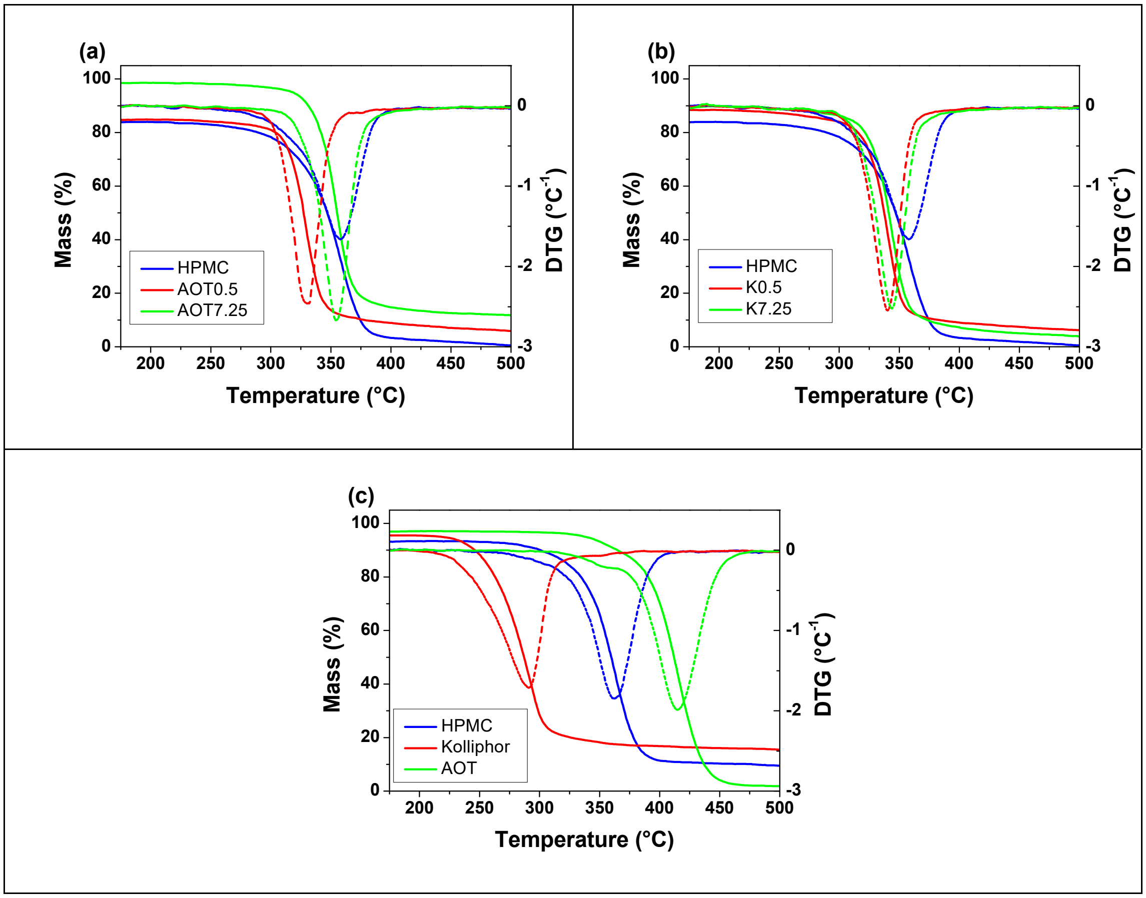

Figure S4. TG/DTG curves from 170-500 °C measured for cryogel samples containing (a) AOT, (b) Kolliphor and (c) pure HPMC (powder), AOT and Kolliphor® EL. The dotted lines represent DTG curves

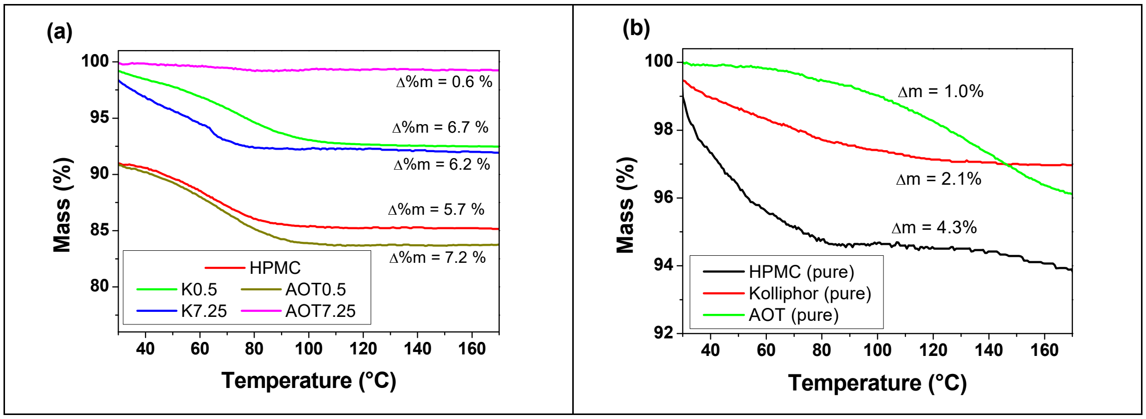

Figure S5. TG curves from 30-170 °C. (a) HPMC, AOT0.5, K0.5, AOT7.25 and K7.25 cryogels and (b) pure HPMC (powder), Kolliphor and AOT.

Sample	Mass of dried sample, m_{pol} (mg)	Mass of sorbed water, m_{water} (g_{water})	Swelling degree, SD (g_{water}/g)
HPMC	43.0 ± 0.1	1.397 ± 0.003	32 ± 1
K0.5	42.3 ± 0.1	1.279 ± 0.002	30 ± 1
K7.25	38.3 ± 0.1	1.226 ± 0.007	32 ± 1
AOT0.5	44.6 ± 0.1	1.287 ± 0.008	29 ± 1
AOT7.25	44.4 ± 0.1	0.971 ± 0.006	22 ± 1

Mass of sorbed MilliQ water (pH 5.5) measured as a function of time at 21.0 ± 0.5 °C with Krüss K100 tensiometer.

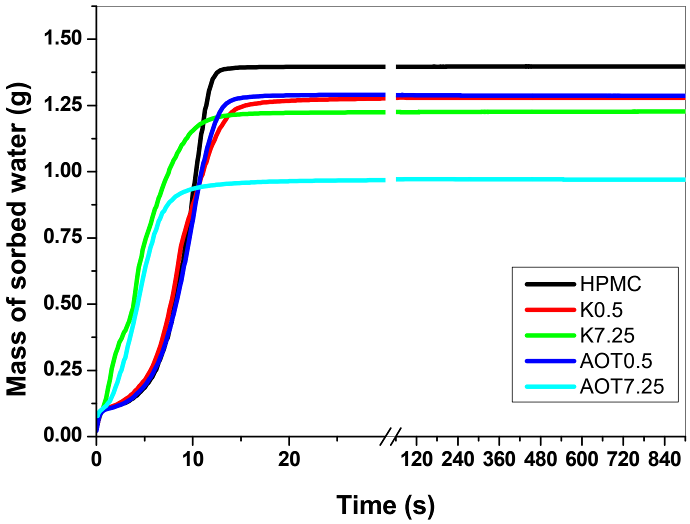

Figure S6. Mass of dried sample, mass of sorbed Milli-Q water and swelling degree (SD) at equilibrium determined for HPMC (blank), and HPMC cryogels prepared with the lowest (K0.5 and AOT 0.5) and highest (K7.25 and AOT7.25) surfactant concentration.

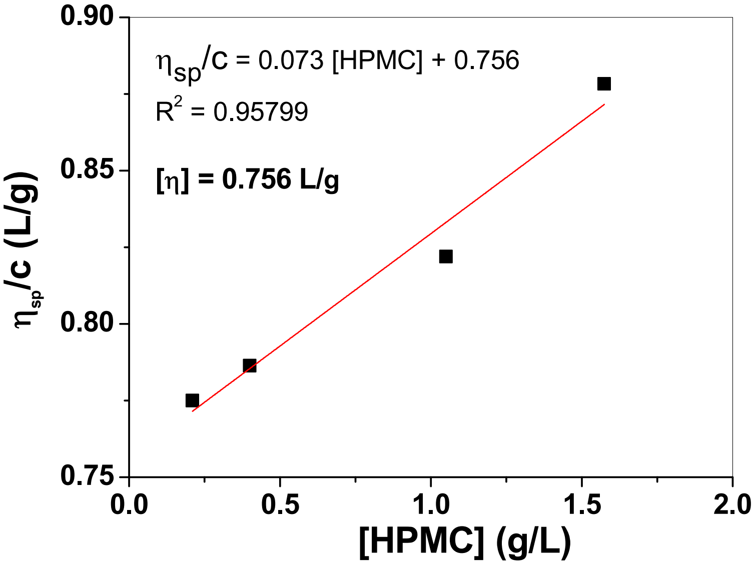

Figure S7. Reduced viscosity η_{sp}/c as a function of HPMC J12MS concentration. Intrinsic viscosity ($[\eta]$) is the intercept of the fitted linear curve. M_v is the viscometric average molar mass and can be obtained by using the Mark-Houwink-Sakurada equation, $[\eta] = KM_v^a$, where K and a are constants. $K = 0.000172$ and $a = 0.838$ for the HPMC-water system [38], resulting in M_v = $3.46\text{x}10^5$ g.mol^{-1}.

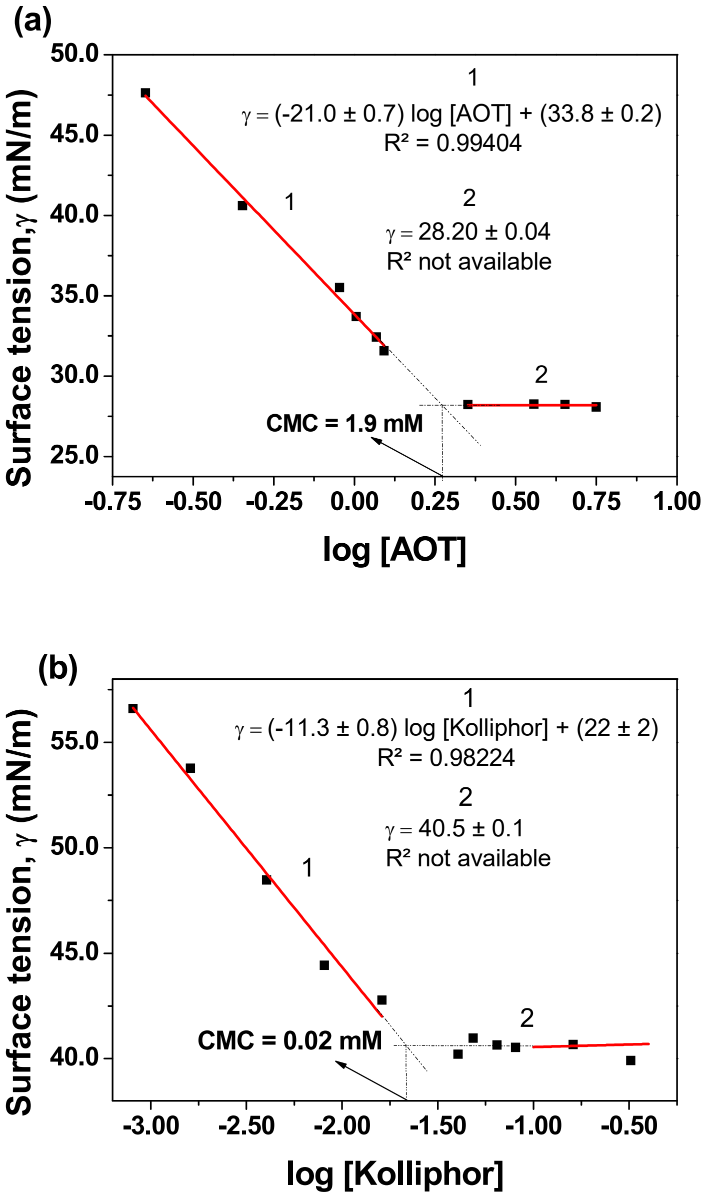

Figure S8. Surface tension (γ) vs logarithm of the concentration (mM) of (a) AOT and (b) Kolliphor® EL aqueous solutions, determined at 21 ± 1 °C, by Du Noüy ring method in a Krüss K100 tensiometer.

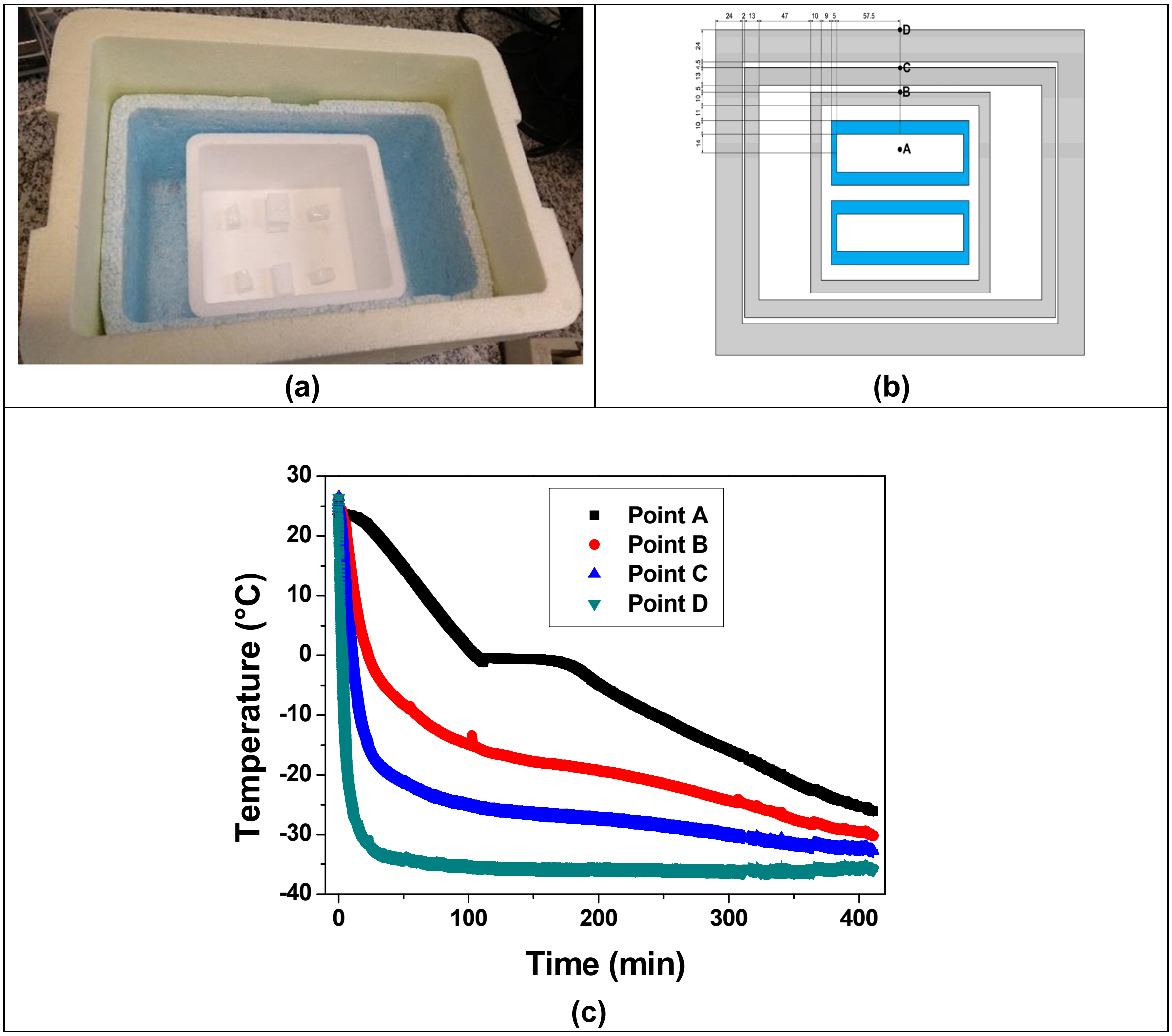

Figure S9. (a) Styrofoam boxes arranged for the precursor gels freezing, (b) top view representation of boxes (gray) with the distances, in millimeters, among the molds (symmetrically positioned in the center) and boxes, as well as their wall thickness and size. (c) The temperature of points A (hydrogel sample in mold A), B, C and D (outer surface of each box) was measured by thermistors as a function of time.

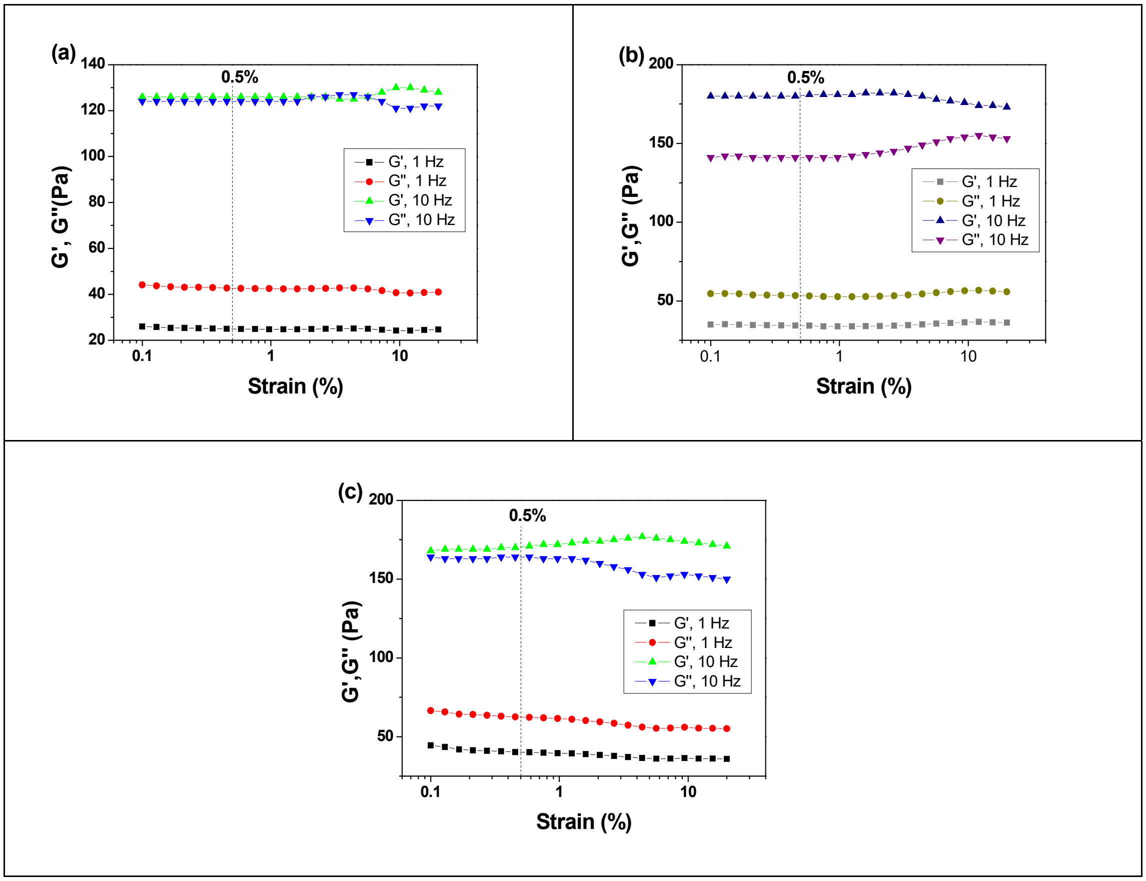

Figure S10. Storage (G') and loss (G'') moduli obtained by DSST tests for hydrogels of (a) HPMC (control), (b) K7.25 and (c) AOT7.25 hydrogels at frequencies of 1 and 10 Hz.

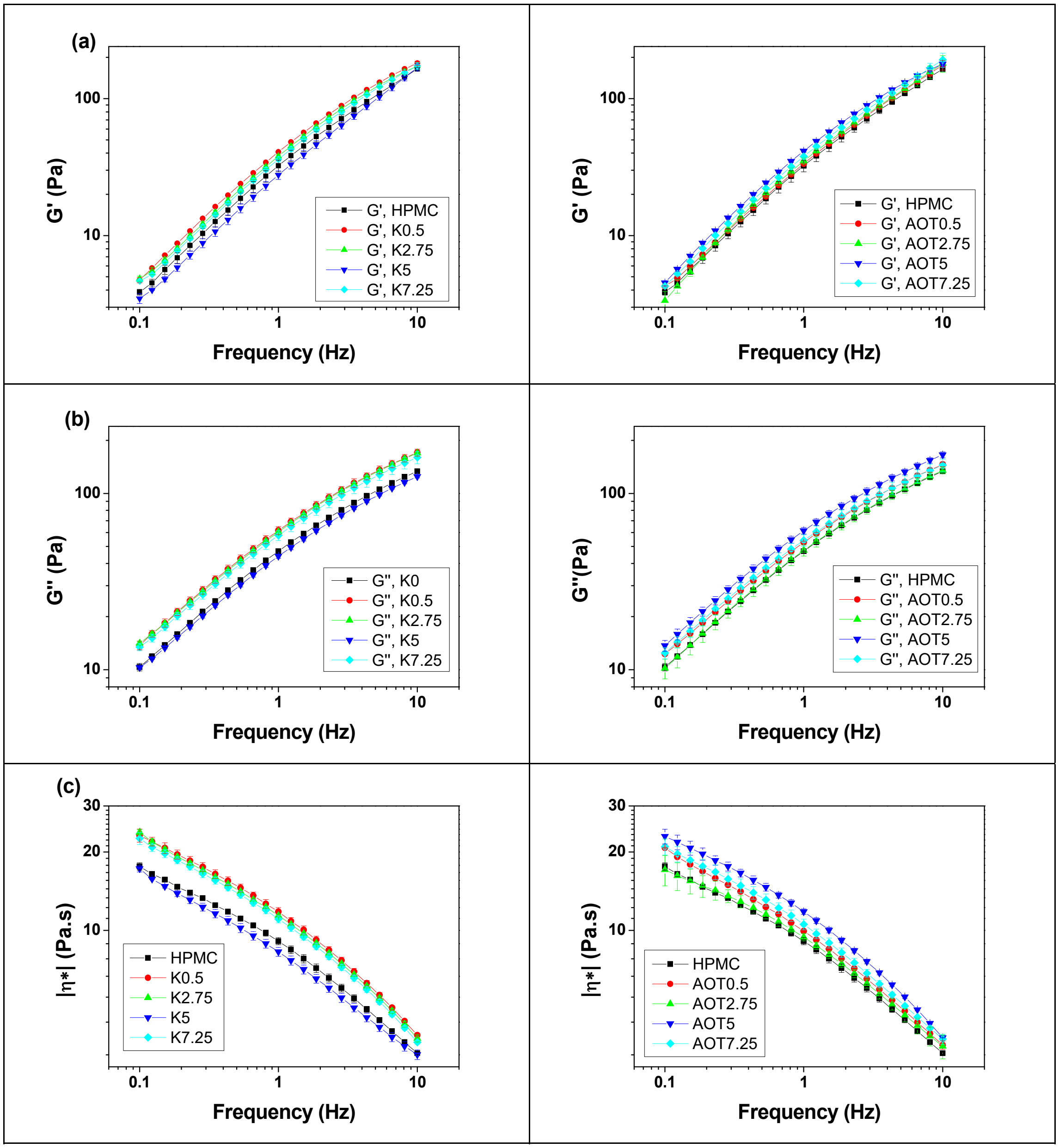

Figure S11. Curves obtained by the SAOS tests performed at (25.0 ± 0.5) °C. (a) Storage modulus, G', (b) loss modulus, G'', (c) G'and G''and (d) complex viscosity, |η*| of HPMC (blank), K0.5, AOT0.5, K2.75, AOT2.75, K5, AOT5, K7.25 and AOT7.25 precursor hydrogels.
